# 55-year-old Woman with Headache, Vomiting, and Visual Disturbance

**DOI:** 10.5811/cpcem.2019.12.45546

**Published:** 2020-04-17

**Authors:** Lana Shaker, Jill Ripper, Tiffany Murano

**Affiliations:** Rutgers New Jersey Medical School, Department of Emergency Medicine, Newark, New Jersey

**Keywords:** headache, pituitary apoplexy, pituitary adenoma

## Abstract

**Case Presentation:**

A 55-year-old woman with a past medical history of hypertension, hyperlipidemia, and iron deficiency anemia presented to the emergency department with three days of headache, nausea, vomiting, and visual changes. Her vital signs were within normal limits. She was noted to have a left cranial nerve six palsy on exam.

**Results:**

Her laboratory testing revealed leukocytosis, hyponatremia, and hypokalemia. A non-contrast computed tomography scan of the head revealed an enlarged sella turcica and pituitary gland with hemorrhage and deviation of the optic chiasm.

**Conclusion:**

Her symptoms improved and she was discharged from the hospital in stable condition.

## CASE PRESENTATION (Lana Shaker)

A 55-year-old woman presented to the emergency department (ED) with the chief complaint of headache for three days, associated with nausea, vomiting, and visual changes. The headache was described as being sudden in onset, constant, bilateral, retro-orbital, and throbbing. The pain was a six out of ten in intensity. The pain was not alleviated with over the counter acetaminophen use. She was not able to describe any alleviating or exacerbating factors. The visual changes were described by the patient as “blurry vision” and “double vision” affecting her left eye greater than her right. She reported photophobia and difficulty keeping the left eye open. The patient described this difficulty of keeping the eye open as a weakness and not secondary to pain. She had approximately five episodes of non-bloody and non-bilious vomiting over the past three days and reported inability to tolerate her home medications. She also reported a sore throat and cough productive of yellow sputum for the previous two to three days, but denied fever, chills, chest pain, hemoptysis, or dyspnea.

Her past medical history included essential hypertension, hyperlipidemia, and a remote history of uterine fibroids associated with iron deficiency anemia. Prescribed medications included losartan 25 milligrams (mg) and hydrochlorothiazide 12.5 mg. She had no known drug allergies, did not smoke, drink alcohol, or use illicit drugs. She was unemployed and lived alone. The patient was post-menopausal and was pregnant three times-two of which were normal spontaneous deliveries with two living children and one prior abortion.

**Image 1:**
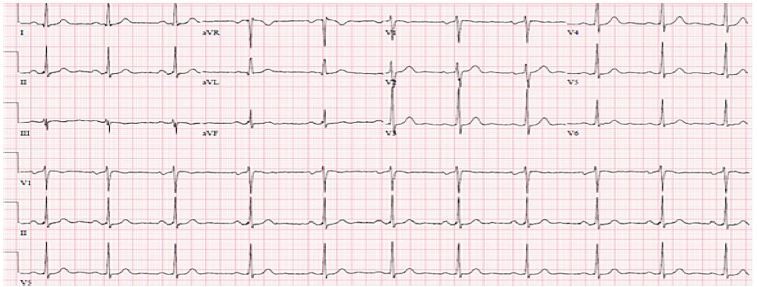
Normal sinus rhythm at 64 beats per minute with sinus arrhythmia.

Vital signs were: temperature 98.8° Fahrenheit, heart rate 84 beats per minute, blood pressure 135/74 millimeters of mercury, respiratory rate of 18 breaths per minute and room air oxygen saturation 97%. Her body mass index was 42 (normal 18.5–24.9). Complete physical examination was unremarkable except her left eye’s lateral gaze was restricted by approximately 25%. Her visual acuity was 20/25 and 20/30, right and left eyes, respectively. Initial laboratory testing were resulted (Tables 1 and 2). An electrocardiogram was performed (Image 1).

## CASE DISCUSSION (Tiffany Murano)

In summary, this is a 55-year-old gravida 3 para 2 woman with a past medical history of hypertensiona and hyperlipidemia who presentes to the ED with a three day history of sudden onset bilateral retro-orbital headache with double vision. She reported a productive cough, which corresponded with the onset of the headache. Her physical examination was significant for restricted lateral gaze of the left eye with an otherwise normal neurologic examination. Her serum laboratory studies demonstrated hyponatremia and leukocytosis.

Headache is the fourth most common chief complaint in the ED and accounts for approximately 3% of ED visits in the United States.^1^ Headaches may be classified as primary (e.g., migraine, tension, cluster headaches). The differential diagnosis for headache is quite broad ranging from benign conditions such as tension headache to potentially life-threatening conditions such as meningitis and stroke. When a patient presents to the ED with a headache, it is important to discern whether the onset of symptoms was progressive or sudden. Symptoms that occur suddenly, as with this patient, often indicates a vascular occlusion (e.g., thrombotic, embolic, or major vessel dissection events) or hemorrhage. The differential, cerebrovascular accident, arteriovenous malformation or a mass with a hemorrhagic component.

Migraine headaches are not generally sudden in onset but can be associated with visual changes, photophobia, nausea, and vomiting. However, the patient has no prior history of migraine headaches, and a new diagnosis of migraine headaches at the age of 55 would be unusual. Optic neuritis can cause acute visual changes and eye pain. However, the visual changes are characterized by decreased visual acuity, visual field loss, photopsia, or color vision loss with pain in the affected eye with ocular movement.^2^ There also may be an afferent pupillary defect. This patient demonstrated none of these findings. Moreover, this disease entity is more commonly in patients with a history of thrombophilia, taking oral contraceptives, pregnant, or post-partum and the majority of patients are under the age of 50 years which makes this diagnosis unlikely in this patient.^3^ Cavernous sinus thrombosis originating from a bacterial sinus infection can present with headache and ocular signs such as orbital pain but is typically not bilateral pain as described by this patient. Additionally, patients with cavernous sinus thrombosis may have proptosis, periorbital erythema and edema, and chemosis on exam, unlike this patient.^4^

The patient has restricted left eye extraocular movements on physical examination with diplopia. She complained of difficulty keeping her left open but had no ptosis noted on physical examination. The sixth cranial nerve (abducens) is responsible for abduction of the lateral rectus muscle that is necessary for horizonto-lateral gaze. Lesions that affect the sixth cranial nerve can cause impairment of lateral gaze as well as Horner’s syndrome (disruption of the sympathetic innervation of the sixth cranial nerve causing miosis, ptosis, and anhidrosis). Etiologies of Horner’s syndrome include pituitary lesions (e.g., infarction or hemorrhage), trauma, brachial plexus lesions, pathology to the lung apice, migraine headache, idiopathic intracranial hypertension, and carotid artery pathology (e.g., ischemia or dissection). However, this patient presented with lateral gaze paralysis in the absence of Horner’s syndrome. Thus, there could be a structural cause-such as a pituitary macroadenoma with or without hemorrhage-preventing normal left lateral gaze.

Another pertinent finding in this case is hyponatremia. Possible explanations for hyponatremia in conjunction with this clinical presentation and neurologic findings include syndrome of inappropriate antidiuretic hormone (SIADH), cerebral salt wasting (CSW), and adrenal insufficiency. Although all present with hyponatremia, CSW is associated with concomitant extracellular volume loss and hypovolemia while SIADH has normal to high extracellular volume and euvolemia. Urine electrolytes were not immediately available for this patient; however, she appeared to be clinically euvolemic. Adrenal insufficiency is an important cause of hyponatremia and may be primary (due to an adrenal cause), secondary (due to an anterior pituitary cause), or tertiary (due to a thalamic cause). Lesions in the pituitary result in a decrease in cortisol, increased adrenocorticotropin hormone (ACTH) and increased corticotropin-releasing hormone secretion (an anti-diuretic hormone secretagogue). Thyroid deficiency also may be seen in central adrenal insufficiency.

In addition, pituitary apoplexy may present with headache, visual disturbance, and hypnoatremia as seen with this patient. The sudden onset of symptoms supports a vascular component-either a hemorrhage or embolic vascular component. A computed tomography scan of the head would be an appropriate initial imaging modality to confirm this diagnosis. According to the American College of Emergency Physicians’ clinical policay for the evaluation and management of adult patients presenting to the ED with an acute headache, there is Level B evidence that supports obtaining an emergency, non-contrast computed tomography (CT) scan of the head for patients with sudden-onset headache and focal neurologic findings. Therefore, this test would be appropriate in this case.^5^

## CASE OUTCOME (Lana Shaker)

**Image 2:**
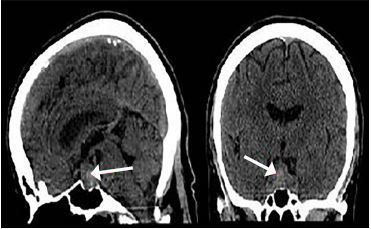
Computed tomography of the head without contrast demonstrating enlarged pituitary gland with increased density consistent with hemorrhage (arrows).

**Image 3:**
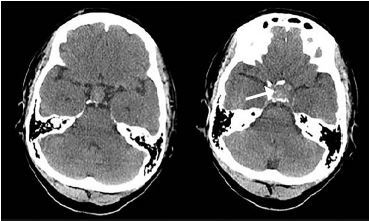
Computed tomography of the head without contrast demonstrating enlarged sella turcica and deviated optic chiasm (arrow).

A non-contrast CT scan of the head revealed an enlarged sella turcica and an enlarged pituitary gland measuring 2.0 centimeters (cm) in its greatest superior to inferior extent, 3.3 cm in its greatest transverse diameter and 1.3 cm in its greatest anterior to posterior extent. In the central and inferior portions of the enlarged pituitary gland, there is increased density consistent with hemorrhage. The floor of the sella turcica is not eroded, and the sphenoid sinus is well aerated. The optic chiasm is deviated superiorly bilaterally (Images 2 and 3). Neurosurgery and ophthalmology services were consulted, and the patient was admitted to the intensive care unit. Her gaze palsy continued to worsen over the subsequent 24 hours (hospital day (HD) 1). Ophthalmology service was consulted and suggested that the patient likely had sixth cranial nerve palsy due to increased intracranial pressure. The neurosurgery team obtained a non-contrast magnetic resonance imaging (MRI) scan of the brain which demonstrated a pituitary macroadenoma displacing the optic chiasm. Additional serum laboratory studies were obtained to assess pituitary function and demonstrated normal thyroid stimulating hormone (TSH), low luteinizing hormone and cortisol levels, and an elevated prolactin level.

On HD 1, the patient also developed worsening hyponatremia and hypotension. She was administered hydrocortisone 100 mg intravenously for a suspected acute ACTH deficiency leading to adrenal crisis. A plan was made for operative intervention. On HD 2 she underwent a trans-sphenoidal surgical resection of the pituitary tumor. Over the subsequent six days, the patient’s headache and visual complaints improved. Her visual acuity improved to 20/25 bilaterally. Her extra-ocular movements normalized with resolution of her cranial nerve six palsy. The hyponatremia resolved. However, she developed central hypothyroidism with both decreasing TSH and free thyroid hormone levels and was administered levothyroxine replacement. The patient was discharged on HD 8. Two week discharge follow up with neurosurgery, ophthalmology, and endocrinology services were unremarkable except for the thyroid hormone supplementation requirement.

## RESIDENT DISCUSSION

Pituitary apoplexy is an acute infarction or hemorrhage of the pituitary gland. In most cases, apoplexy involves a previously unrecognized pituitary adenoma.^4^ An abrupt increase of tissue volume within the sellar region can cause headache, visual impairment, cranial nerve palsies, impairment of consciousness, and pituitary hormone deficiencies.^4^ Pituitary apoplexy is rare with an estimated incidence of 0.17 episodes per 100,000 person-years but is life threatening and must be promptly recognized and treated.^4^ Most commonly, pituitary apoplexy occurs in the fifth or sixth decade and has a slight male preponderance.^4^,^5^ Pituitary apoplexy can occur in non-adenomatous lesions including: hypophysitis, pituitary metastasis, craniopharyngioma, Rathke’s cleft cyst, and sellar tuberculoma.^4^ Macroadenomas are more susceptible to apoplexy than microadenomas.^6^ Between 2 and 12% of patients with a pituitary adenoma experience apoplexy.^7^ Cavernous sinus invasion can be prognostic factor associated with pituitary apoplexy.^6^

The mechanisms causing pituitary apoplexy include: tumor vascular occlusion due to tumor growth, pituitary stimulation (e.g., provocative testing or gonadotropin releasing hormone analogue use, surgery, closed trauma, acute increase in blood flow due to physical activity or systemic hypertension, or coagulation disturbances (e.g., thrombocytopenia or anticoagulation).^6^,^7^ Macroprolactinomas and female gender have a greater association with hemorrhage.^4^

Symptoms may arise within hours to days after the onset of apoplexy.^4^ Sudden increase in intrasellar pressure can cause hypopituitarism. Moreover, sudden increases in pressure on contents and in neural structures can cause: neural palsies (most commonly cranial nerves III, IV, V, or VI), visual field impairment and visual acuity deficiency (due to optic chiasm compression), consciousness reduction (due to pressure transmitted to the brainstem), chemical meningitis (from blood leakage into the subarachnoid space), and even hemispheric signs such as hemiplegia (from intracavernous carotid artery compression and vasospasm).^6^

A high degree of suspicion is required to make the clinical diagnosis as these patients typically do not have a known history of pituitary disease.^4^ Common differential diagnoses include subarachnoid hemorrhage, meningitis, cavernous sinus thrombosis, and migraine.^4^ A CT scan of the head is more pragmatic to obtain but less sensitive to diagnose a pituitary lesion than brain MRI.^6^,^8^

Hormonal pituitary evaluation is recommended as anterior pituitary deficiencies can occur in nearly 80% of patients.^6^ ACTH deficiency can lead to adrenal crisis and is life threatening, requiring immediate glucocorticoid replacement.^5^ Other deficiencies, such as hyperprolactinemia or hypothyroidism, may also occur.^8^ Hyponatremia is observed in up to 40% of cases and is due to decreased circulating cortisol in the setting ACTH deficiency or SIADH.^6^

The first step in management of patients with pituitary apoplexy is hemodynamic stabilization. Patients may require correction of electrolye disturbances and corticosteroid administration. Further management may be either surgical or continued medical care, and some controversy exists regarding this issue.^10^ If consciousness or vision is impaired, surgical decompression is recommended.^6^,^10^ Pituitary deficiencies, however, do not generally recover.^6^ Outcome is variable and difficult to predict as patients may dramatically deteriorate from cerebral ischemia or subarachnoid hemorrhage or recover spontaneously without sequelae.^7^

## FINAL DIAGNOSIS

Pituitary macroadenoma with apoplexy.

## KEY TEACHING POINTS

- Pituitary apoplexy can evolve in hours to days and is life threatening.
- Pituitary apoplexy is rare, most common in the fifth and sixth decade, and has a slight male preponderance. Pituitary adenomas, specifically macroadenomas, are at highest risk of apoplexy and is often a new diagnosis.
- The pathophysiology regarding pituitary apoplexy is associated with hypertension, coagulopathy, recent surgery or closed head trauma.
- Symptoms and signs include: acute headache, nausea and vomiting, visual disturbances, ocular palsies, meningismus, and decreased level of consciousness.
- Head CT should be obtained immediately but brain MRI is most sensitive for the diagnosis of pituitary apoplexy and should be obtained if clinical suspicion is high despite non-diagnostic CT
- Treatment is comprised of circulatory support, electrolyte correction, corticosteroid administration when indicated, and potential surgical decompression.
- Outcome is variable, ranging from death or persistent neurological sequelae to spontaneous recovery.

## Figures and Tables

**Table 1:** Complete blood cell count.

Serum hematology test	Value (reference range)
Complete blood cell count	
White blood cells	14.9 × 10^3^/uL (4.0–11.0)
Hemoglobin	14 g/dL (12.0–16.0)
Hematocrit	43% (36.0–48.0)
Platelets	225 × 10^3^/uL (150–450)
Differential	
Neutrophils	78% (35.0–80.0)
Lymphocytes	13% (20.0–50.0)
Monocytes	7% (2.0–12.0)
Eosinophils	0.6% (0.0–7.0)
Basophils	0.8% (0.0–2.0)

*uL*, microliters; *g*, grams; *dL*, deciliter.

**Table 2:** Chemistry results.

Serum chemistry test	Value (reference range)
Complete metabolic panel	
Sodium	128 mmol/L (133–145)
Potassium	3.4 mmol/L (3.5–4.8)
Chloride	88 mmol/L (97–110)
Bicarbonate	27 mmol/L (23–30)
Blood urea nitrogen	7 mg/dL (6–20)
Creatinine	0.7 mg/dL (0.5–1.0)
Glucose	81 mg/dL (70–109)
Albumin	4.0 g/dL (3.5–5.2)
Bilirubin	1.0 mg/dL (<= 1.0)
Alkaline phosphatase	87 U/L (35–105)
Total protein	8.5 g/dL(6.0–8.3)
Aspartate transaminase	18 U/L (0–40)
Alanine aminotransferase	11 U/L (0–33)
Additional chemistries	
Troponin	<0.01 ng/mL (0.00–0.30)

*mmol*, millimoles; *L*, liter; *mg*, milligram; *dL*, deciliter; *U*, units; *ng*, nanogram; *mL*, milliliter.
